# Macrophage malfunction in Triptolide-induced indirect hepatotoxicity

**DOI:** 10.3389/fphar.2022.981996

**Published:** 2022-09-26

**Authors:** Tingting Qin, Muhammad Hasnat, Yang Zhou, Ziqiao Yuan, Wenzhou Zhang

**Affiliations:** ^1^ Department of Pharmacy, The Affiliated Cancer Hospital of Zhengzhou University and Henan Cancer Hospital, School of Pharmaceutical Sciences, Zhengzhou University, Zhengzhou, China; ^2^ Key Laboratory of Advanced Drug Preparation Technologies, Ministry of Education, School of Pharmaceutical Sciences, Zhengzhou University, Zhengzhou, China; ^3^ Henan Engineering Research Center for Tumor Precision Medicine and Comprehensive Evaluation, The Affiliated Cancer Hospital of Zhengzhou University and Henan Cancer Hospital, Zhengzhou University, Zhengzhou, China; ^4^ Henan Provincial Key Laboratory of Anticancer Drug Research, The Affiliated Cancer Hospital of Zhengzhou University and Henan Cancer Hospital, Zhengzhou University, Zhengzhou, China; ^5^ Institute of Pharmaceutical Sciences, University of Veterinary and Animal Sciences, Lahore, Pakistan; ^6^ Children’s Hospital Affiliated to Zhengzhou University, Henan Children’s Hospital, Zhengzhou Children’s Hospital, Zhengzhou University, Zhengzhou, China

**Keywords:** Triptolide, LPS, MERTK, hepatic macrophage, hypersensitization

## Abstract

**Background and Objective:** Indirect hepatotoxicity is a new type of drug-induced hepatotoxicity in which the character of a drug that may induce its occurrence and the underlying mechanism remains elusive. Previously, we proved that Triptolide (TP) induced indirect hepatotoxicity upon LPS stimulation resulting from the deficiency of cytoprotective protein of hepatocyte. However, whether immune cells participated in TP-induced indirect hepatotoxicity and the way immune cells change the liver hypersensitivity to LPS still need to be deeply investigated. In this study, we tried to explore whether and how macrophages are involved in TP-induced indirect hepatotoxicity.

**Method:** Firstly, TP (500 μg/kg) and LPS (0.1 mg/kg) were administrated into female C57BL/6 mice as previously reported. Serum biochemical indicators, morphological changes, hepatic macrophage markers, as well as macrophage M1/M2 markers were detected. Secondly, macrophage scavenger clodronate liposomes were injected to prove whether macrophages participated in TP-induced indirect hepatotoxicity. Also, the ability of macrophages to secrete inflammatory factors and macrophage phagocytosis were detected. Lastly, reverse docking was used to find the target of TP on macrophage and the possible target was verified *in vivo* and in RAW264.7 cells.

**Results:** TP pretreatment increased the liver hypersensitization to LPS accompanied by the recruitment of macrophages to the liver and promoted the transformation of macrophages to M1 type. Depletion of hepatic macrophages almost completely alleviated the liver injury induced by TP/LPS. TP pretreatment increased the secretion of pro-inflammatory factors and weakened the phagocytic function of macrophages upon LPS exposure. Reverse docking results revealed that MerTK might be the real target of TP.

**Conclusion:** TP disrupts inflammatory cytokines profile and phagocytic function of hepatic macrophages, resulting in the production of massive inflammatory factors and the accumulation of endotoxin in the liver, ultimately leading to the indirect hepatotoxicity of TP. MerTK might be the target of TP on the macrophage, while the binding of TP to MerTK should be investigated *in vivo* and *in vitro*.

## Introduction

Typically, drug-induced liver injury was classified into direct or idiosyncratic. Direct hepatotoxicity is caused by the intrinsic toxicity of the agents (Acetaminophen, Aspirin, and Chemotherapy agents) (Chen et al., 2019). The agents (Amoxicillin–clavulanate, Isoniazid, Polygonum multiflorum) that may cause idiosyncratic hepatotoxicity usually have low cytotoxicity to liver cells, and the occurrence of idiosyncratic hepatotoxicity results from patients themselves, such as HLA mutation (Hoofnagle and Bjornsson, 2019). Recently, a new type of drug-induced liver injury, indirect hepatotoxicity, was proposed (Hoofnagle and Bjornsson, 2019). In contrast to direct and idiosyncratic hepatotoxicity, indirect hepatotoxicity was caused by the action of agents on the liver or immune system and is tightly related to the pharmacological effects of the agents. Indirect toxicity is gradually recognized by researchers while its mechanisms have not been elucidated.

Triptolide (TP), the main compound derived from the traditional Chinese medicine *Tripterygium wilfordii Hook F*., possesses strong bioactivities for the treatment of tumor and autoimmune diseases, especially rheumatoid arthritis, systemic lupus erythematosus, and nephrotic syndrome (Li et al., 2014). As the major active and toxic compound of Tripterygium wilfordii multiglycoside in clinical practice, most of the published research articles focused on the direct hepatotoxicity induced by the high dose of TP (Wang et al., 2014; Hasnat et al., 2019a; Yuan et al., 2019a; Hasnat et al., 2019b). Previously, we proposed a new perspective of TP-associated hepatotoxicity, liver hypersensitization upon Lipopolysaccharide (LPS) treatment (Yuan et al., 2019b). Additionally, we proved that TP pretreatment increased the sensitivity of hepatocytes to TNF-α stimulation, which was mainly released by macrophages upon LPS treatment. A mechanistic study uncovered that the inhibition of c-FLIP (cellular FLICE-like inhibitory protein) by TP resulted in hepatocytes’ hypersensitization upon TNF-α stimulation (Yuan et al., 2020). These results implied the crosstalk between immune cells, especially macrophages which are the main source of TNF-α, and hepatocytes in TP/LPS-induced liver injury. Thus, identification of the functional changes in immune cells and exploring the key mechanisms will be of great significance to elucidate the pathogenesis of TP-induced indirect hepatotoxicity.

LPS, a component of the outer cell wall of gram-negative bacteria, is the ligand of TLR4 on the surface of macrophages. The binding of LPS on TLR4 activates TRIF and Myd88 pathway, ultimately leading to the production of pro-inflammatory factors, such as IL-1, IL-6, TNF-α, IL-8, and others (Skirecki and Cavaillon, 2019). Liver macrophages, which function as scavenging bacteria and microbial products along with initiating or suppressing the immune response, can be classified into two populations and play different roles in liver immune homeostasis (Krenkel and Tacke, 2017). CD11b^+^ macrophages derived from infiltrating monocytes of blood functioned as the production of massive inflammatory factors. While liver-resident macrophages expressed low CD11b^low^ (also known as Kupffer cells), maintaining homeostasis of the liver via phagocytosis, cleaning of cellular debris, regulation of cholesterol homeostasis, and mediation of anti-microbial defense (Ju and Tacke, 2016; Wen et al., 2021). Macrophage malfunction has been proved to participate in NAFLD, liver fibrosis, hepatocellular carcinoma, and acetaminophen-induced acute hepatotoxicity. Also, modulation of macrophage polarization/reprogramming, inhibition of Kupffer cell activation, and dampening of monocyte recruitment serve as the principal patterns for treating macrophage-related liver disease (Tacke, 2017; van der Heide et al., 2019). Targeting macrophage ASK1 (ASK1 inhibitor Selonsertib), CCR5 (CCR5 antagonist Maraviroc), CCR2 (CCR2/5 due-inhibitor Cenicriviroc), and CSF1 (CSF1R antibody and inhibitor) have been proved to have a good therapeutic effect on acute liver failure. NASH, liver fibrosis, HIV, and liver cancer (Marra and Tacke, 2014; Stutchfield et al., 2015; Lefebvre et al., 2016; Tacke, 2017; Challa et al., 2019). Recently, a new macrophage efferocytosis receptor c-mer tyrosine kinase (MerTK) was proposed to be an ideal target for liver disease treatment. The activation of TLR receptors could initial the activation of TRAF3 and TRAF6, which in turn activate NF-κB and IRF3, leading to the production of inflammatory factors and type I interferons. Also, interferon signaling further stimulates the production of interferon via STAT1 and upregulates the TAM receptors (Tyro3/Axl/MerTK) (O'Neill, 2007). It has been proved that macrophage MerTK activated HSCs and induced liver fibrosis via ERK-TGFβ1 pathway (Cai et al., 2020). Macrophage MerTK also relieved liver inflammation in acute liver failure (Cai et al., 2016; Cai et al., 2018). Additionally, MerTK inhibitor augmented the anti-PD-1 therapy in cancer cells (Holtzhausen et al., 2019; Kasikara et al., 2019). Mechanistic studies revealed that the bio-activities of MerTK depended on its effect on the secretion of inflammatory factors and phagocytic functions of macrophages (Rothlin et al., 2007; Graham et al., 2014; Vago et al., 2021).

Considering the crucial role of TNF-α in TP-induced indirect hepatotoxicity and inflammatory secretions by hepatic macrophages, we proposed that the function of hepatic macrophages might be disrupted by TP, leading to the massive production of TNF-α and abnormal accumulation of LPS in the liver. The abundant TNF-α coordination along with the deficiency of the pro-survival signal induced by TP as reported in our previous research led to liver injury accompanied by hepatocyte apoptosis and necrosis (Yuan et al., 2020). In this study, the abundance of hepatic macrophages, phagocytosis, and inflammatory secretions by hepatic macrophages were detected to testify the functional changes in macrophages after TP/LPS treatment. Moreover, the clodronate liposomes were injected to deplete hepatic macrophages and to prove whether hepatic macrophages participated in TP-induced indirect hepatotoxicity. Additionally, reverse docking was used to predict the possible target of TP in macrophages and MerTK expression was verified *in vivo* and *in vitro*.

## Materials and methods

### Material

TP (CAS number 38748-32-2, purity >98%) was purchased from MedChemExpress (Shanghai, China). LPS (L2755) was purchased from Sigma-Aldrich (St. Louis, MO, United States). Trizol reagent used for RNA isolation, Reverse Transcription Kit used for reverse transcription of RNA to cDNA, and SYBR Green Master Mix used for qPCR, were obtained from Vazyme Biotech Co., Ltd. (Nanjing, Jiangsu, China). Mouse recombinant Growth arrest-specific protein 6 (GAS6, 8310-GS) was purchased from R&D Systems, Inc. (Minneapolis, MN, United States).

Primary antibodies against p-MerTK (ab14921) and cleaved caspase-3 (9661) were purchased from Abcam (Cambridge, United Kingdom) and Cell Signaling Technology (Boston, MA, United States) respectively. Antibodies against GAPDH (10494-1-AP), Myeloperoxidase (MPO, 22225-1-AP), CD11b (21851-1-AP), and F4/80 (28463-1-AP) were purchased from Proteintech (Chicago, IL, United States). HRP Conjugated AffiniPure Goat Anti-rabbit (BA1054) and HRP Conjugated AffiniPure Goat Anti-mouse antibodies were purchased from Boster Biological Technology Co.Ltd. (Wuhan, Hubei, China). ELISA kits of IL-1β (KE10003), TNF-α (KE10002), and IL-6 (KE10007) were purchased from Proteintech. The endotoxin detection kit (EC80545S) was purchased from Bioendo Technology Co., Ltd. (Xiamen, Fujian, China). Clodronate liposomes and its negative control were purchased from Yeasen Biotechnology Co., Ltd. (Shanghai, China).

### Animal and pharmacological treatments

Animal treatment was described in our previous research (Yuan et al., 2019b; Yuan et al., 2020; Zhang et al., 2022). C57BL/6 mice used in this experiment were purchased from SPF (Beijing) Biotechnology Co., Ltd. Briefly, TP (500 μg/kg, i.g.) was administered daily through gavage for 7 days followed by a single dose of LPS (0.1 mg/kg, i.p.) 2 h after the last dose of TP into female C56BL/6 mice (6–8 weeks old). The phagocytic function and level of hepatic macrophages were determined 6 h after LPS injection. Serum inflammatory factors were detected 1 h, 3 h, and 6 h after LPS injection, respectively.

To prove the involvement of hepatic macrophages in TP/LPS induced hepatotoxicity, Clodronate liposomes (200 μL/mouse, i.p.) and its negative control were injected 48 h before LPS administration to deplete macrophages (Zeng et al., 2020). Mice were sacrificed 6 h after LPS treatment and blood, as well as liver samples, were collected for further examination. All experimental procedures involving mice were complied with the ARRIVE guidelines and were permitted by the Animal Ethics Committee, Zhengzhou University.

### Blood biochemistry analysis

After collection, blood samples were stored at room temperature for 1 h and then centrifuge for 10 min at 3500 rpm/min. Serum was collected for blood biochemistry analysis. Serum alanine aminotransferase (ALT), aspartate aminotransferase (AST), albumin (ALB), alkaline phosphatase (ALP), and total protein were detected using kits from Weiteman Biotech (Nanjing, Jiangsu, China). Serum total bile acid (TBA) was determined using the kit from Jiancheng Bioengineering Institute (Nanjing, Jiangsu, China). All kits were used according to the manufacturer’s instructions.

### Histopathological examination

Liver sections were collected and fixed with 10% formaldehyde for 24 h. After embedding in paraffin and slicing with a tissue sectioner, liver sections were used for hematoxylin and eosin (H&E) staining to examine the morphology. Additionally, immunohistochemistry (IHC) staining of MPO, F4/80, CD11b, and cleaved caspase-3 were used to determine inflammatory infiltration, macrophage number, and apoptosis rate of hepatocytes respectively. All the slices were observed with ANNORAMIC MIDI II (3DHISTECH Ltd., Budapest, Hungary).

### Cell culture

The mouse monocyte/macrophage cell line RAW264.7 was purchased from China Cell Culture Center (Shanghai, China). The cell was cultured in DMEM with 10% FBS at 37°C in a humidified 5% CO_2_ atmosphere. Cells were seeded into the 6-well plate to determine the effect of TP (20 nmol/L) and LPS (50 ng/ml) on p-MerTK expression. When the cells reached 40%–50% confluence, TP was added to the medium. Twenty-4 hours after TP treatment, the cell pellet was collected for further detection. To investigate the effect of TP on *SOCS1* (Suppressor Of Cytokine Signaling 1) and *SOCS3* (Suppressor Of Cytokine Signaling 3) expression after Gas6, cells were seeded into the 6-well plate. When the cells reached 80%–90% confluence, TP (20 nmol/L) was added 1 h before Gas6 (50 nmol/L). 30 min, 60 min, 90 min, and 120 min after Gas6 exposure, cells were collected for further detection.

### RNA extraction and qPCR

A total of 50 mg liver tissue was separated from liver samples stored at −80°C and RNA was extracted with TRIzol reagent. After the quantification with Nanodrop 3000 (Thermo Fisher Scientific, Waltham, MA, United States), 1 μg of total RNA of each sample was reverse transcribed to cDNA with Reverse Transcription Kit. The relative mRNA expression of macrophage markers and inflammatory factors were determined by QuantStudio six Flex (Thermo Fisher Scientific) with SYBR Green Master Mix and normalized with *Gapdh*. The primers used in this paper were listed in Table 1.

**TABLE 1:** The primer sequences used for qPCR assay in mice.

Gene	Forward primer (5′-3′)	Reverse primer (5′-3′)
*Gapdh*	CAT​CAC​TGC​CAC​CCA​GAA​GAC​TG	ATG​CCA​GTG​AGC​TTC​CCG​TTC​AG
*Adgre1*	CGT​GTT​GTT​GGT​GGC​ACT​GTG​A	CCA​CAT​CAG​TGT​TCC​AGG​AGA​C
*Itgam*	TAC​TTC​GGG​CAG​TCT​CTG​AGT​G	ATG​GTT​GCC​TCC​AGT​CTC​AGC​A
*Cd68*	GGC​GGT​GGA​ATA​CAA​TGT​GTC​C	AGC​AGG​TCA​AGG​TGA​ACA​GCT​G
*Il12b*	TTG​AAC​TGG​CGT​TGG​AAG​CAC​G	CCA​CCT​GTG​AGT​TCT​TCA​AAG​GC
*Nox2*	GAG​ACA​GGG​AAG​TCT​GAA​GCA​C	CCA​GCA​GTA​GTT​GCT​CCT​CTT​C
*Il1b*	TGG​ACC​TTC​CAG​GAT​GAG​GAC​A	GTT​CAT​CTC​GGA​GCC​TGT​AGT​G
*Mrc1*	GTT​CAC​CTG​GAG​TGA​TGG​TTC​TC	AGG​ACA​TGC​CAG​GGT​CAC​CTT​T
*Arginase1*	CAT​TGG​CTT​GCG​AGA​CGT​AGA​C	GCT​GAA​GGT​CTC​TTC​CAT​CAC​C
*Retnla*	CAA​GGA​ACT​TCT​TGC​CAA​TCC​AG	CCA​AGA​TCC​ACA​GGC​AAA​GCC​A
*SOCS1*	AGT​CGC​CAA​CGG​AAC​TGC​TTC​T	GTA​GTG​CTC​CAG​CAG​CTC​GAA​A
*SOCS3*	GGA​CCA​AGA​ACC​TAC​GCA​TCC​A	CAC​CAG​CTT​GAG​TAC​ACA​GTC​G

### Western blot analysis

A total of 100 mg liver protein was used for the protein extraction with RIPA Lysis Buffer (Solarbio Science & Technology Co., Ltd., Beijing, China). After quantification with BCA kit (Solarbio Science & Technology Co.,Ltd.), the protein was mixed with a 4×loading buffer containing protease and phosphatase inhibitor cocktail (P1049, Beyotime Biotechnology, Shanghai China). The same amount of protein was added to each well for SDS-PAGE with gels ranging from 10% to 12%. The protein was then transferred onto nitrocellulose filter membrane (Pall Corporation, NY, United States). The membranes were then incubated with secondary antibodies for 1 h and visualized at Tanon-5200 Chemiluminescent Imaging System (Tanno, Shanghai, China) using an ECL detection kit (Millipore, Danvers, MA, United States). The relative protein expression was normalized with GAPDH and analyzed with ImageJ 1.52a (Bethesda, MD, United States).

### Molecular docking

Autodock Vina 1.1.2 (The Scripps Research Institute, La Jolla, CA United States) was used to analyze the binding of TP to MerTK. Briefly, TP was docked into the crystal structure of human TAK1 ligand-binding domains (RCSB code: 7VAX) under default parameters. TP-MerTK interaction was calculated with Autodock Tools 1.5.6 and visualized with PyMOL 2.5.0. The details of our steps are present in the previous research (Zhao et al., 2019).

### Statistical analysis

Data of this research were analyzed by GraphPad Prism 8.0.1. (GraphPad Software, San Diego, CA, United States). All the data are presented in Mean ± SEM. One-way ANOVA followed by Tukey’s Multiple Comparison Test was performed to analyze the differences between groups. *p*-values < 0.05 were considered to be statistically significant.

## Result

### TP-treatment increased the sensitivity of the liver to LPS administration

**FIGURE 1:**
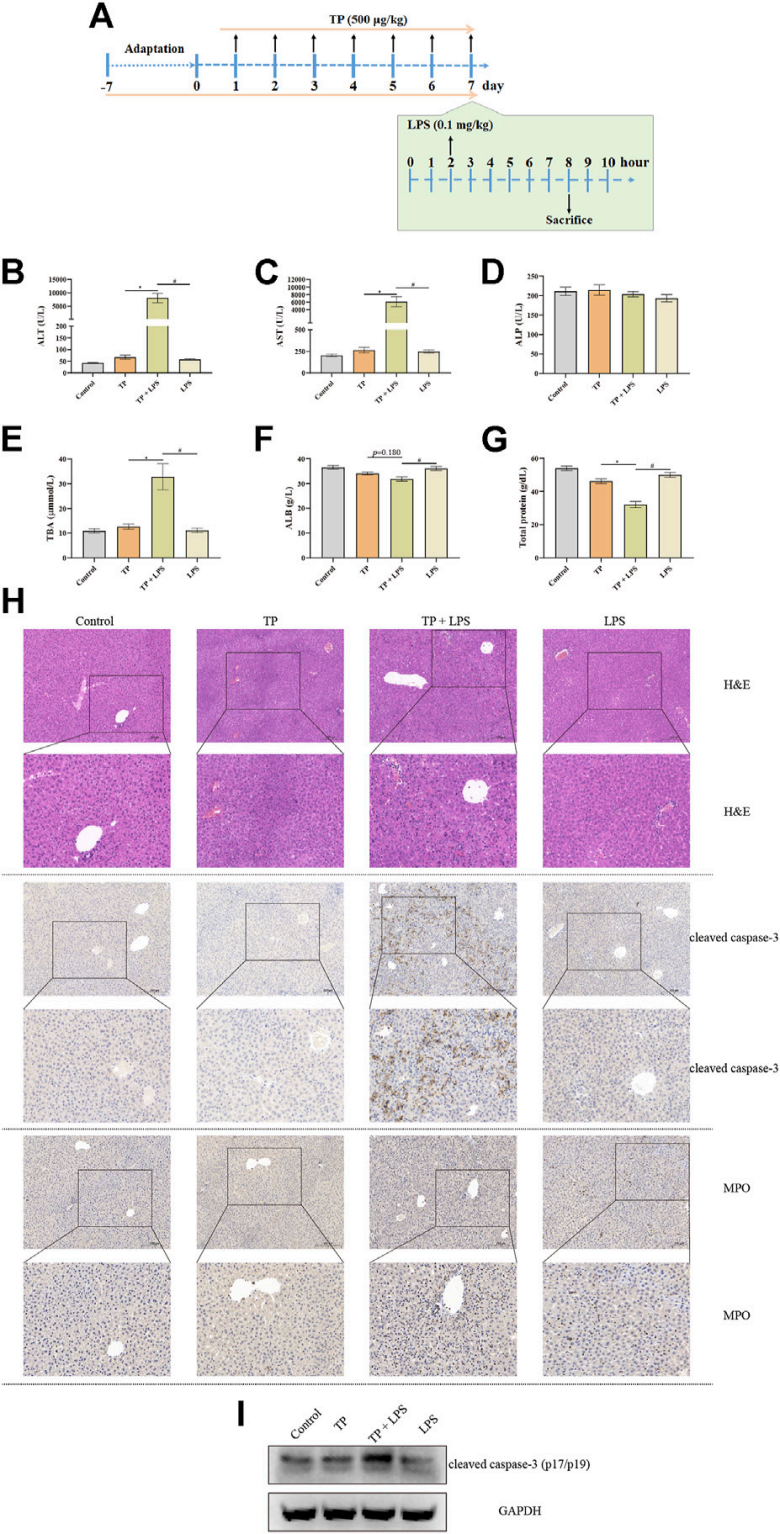
TP treatment increased the sensitivity of the liver to LPS administration. **(A)** Schematic presentation of the experimental procedure to prove the hypersensitivity of TP (500 μg/kg) in pretreated mice to LPS (0.1 mg/kg). **(B–G)** Levels of serum ALT, AST, ALP, TBA, ALB, and total protein in mice treated with TP and LPS (*n* = 8). **(H)** Pictures of liver tissue sections stained by H&E (top panels), analyzed by IHC for MPO (middle panels), or analyzed by cleaved caspase-3 (bottom panels) (×200). **(I)** Representative protein bands of cleaved caspase-3 after TP/LPS treatment, with GAPDH as the loading control. Results were expressed as means ± SEM. Statistical analysis was performed using One-way ANOVA followed by Tukey’s Multiple Comparison Test. *p* < 0.05 was considered to be statistically different.

Although TP, as well as Tripterygium wilfordii multiglycoside, the fat-soluble mixture refined from *Tripterygium wilfordii Hook F.* commonly used for the treatment of rheumatoid arthritis (RA) and immune diseases in clinical practice, might induce severe liver injury as observed in clinical report, our previous research proved that the direct hepatotoxicity of TP might not be the main reason for TP-induced hepatotoxicity (Li et al., 2014). Additionally, we proposed that the indirect toxicity of TP might be the main reason for TP-induced hepatotoxicity. In this study, TP (500 μg/kg) was administrated continuously for 7 days followed by a single dose of LPS (0.1 mg/kg) (Figure 1A). Six hours after LPS stimulation, serum and liver samples were collected for further detection. We found that TP and LPS co-treatment significantly increased serum ALT and AST, the two transaminases reflecting the degree of the liver damage, while TP and LPS alone had little effect on ALT and AST (Figures 1B,C). Moreover, TP/LPS co-treatment increased the serum TBA levels while did not affect serum ALP, reflecting that TP/LPS co-treatment might not attract bile duct cells (Figures 1D,E). Serum ALB and Total protein were also decreased by TP/LPS co-treatment, implying TP/LPS might disrupt liver protein synthesis (Figures 1F,G). Liver H&E staining revealed that TP/LPS co-treatment promoted hepatic cell apoptosis and necrosis, which was further proved by IHC staining and western blot of cleaved caspase-3 (Figures 1H,I). Moreover, TP/LPS co-treatment promoted the recruitment of inflammatory cells, as reflected by the IHC staining of MPO (Figure 1H). Overall, our results proved that TP pretreatment increased the sensitivity of the liver to LPS, accompanied by inflammatory cell infiltration. However, the actual role of immune cells in TP/LPS-induced indirect hepatotoxicity still needs to be investigated.

### TP-induced indirect hepatotoxicity accompanied by macrophage recruitment and fostered the polarization of macrophages to M1 type

**FIGURE 2:**
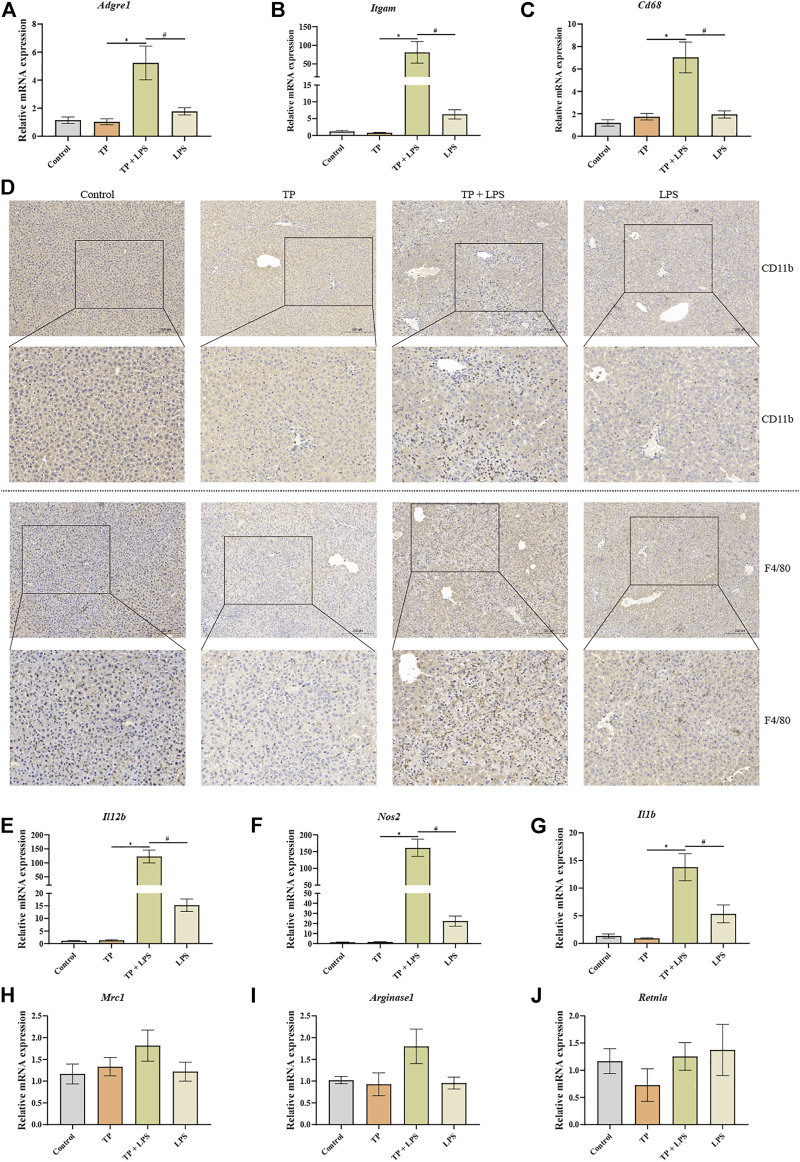
TP-induced indirect hepatotoxicity accompanied by macrophage recruitment and fostered the polarization of macrophages to M1 type. **(A–C)** The relative mRNA levels of hepatic macrophage markers *Adgre1*, *Itgam*, and *Cd68* were detected by qPCR with *Gapdh* as the internal control (*n* = 8). **(D)** Pictures of liver tissue sections analyzed by IHC for CD11b (top panels) and F4/80 (bottom panels) (×200). **(E–J)** The relative mRNA levels of M1 macrophage markers (*IL12b*, *Nos2*, and *Il1b*) and M2 macrophage markers (*Mrc1*, *Arginase1*, and *Retnla*) after TP/LPS treatment, with *Gapdh* as the internal control (*n* = 8). Results were expressed as means ± SEM. Statistical analysis was performed using One-way ANOVA followed by Tukey’s Multiple Comparison Test. *p* < 0.05 was considered to be statistically different.

Liver macrophages, the key cellular component of liver non-parenchymal cells, are essential to maintain liver homeostasis and promote or regress liver injury (Krenkel and Tacke, 2017). We speculated that liver macrophages might promote liver injury after TP/LPS co-treatment. To prove this, we detected the number of macrophages with IHC staining and qPCR. The results of qPCR revealed that macrophage markers *Adgre1* (encoding F4/80), *Itgam* (encoding CD11b), and *Cd68* were substantially increased in TP/LPS co-treatment group (especially *Itgam*), while TP and LPS alone had little effect on the expressions of *Adgre1* and *Cd68*. Additionally, LPS slightly increased the expression of *Itgam* (Figure 2A–C). The IHC staining of F4/80 and CD11b also firmly confirmed the qPCR results (Figure 2D). Hepatic macrophages can be divided into two parts, liver resident macrophages (also known as Kupffer cells) originating from erythromyeloid progenitors of the yolk sac expressing CD68 and blood-derived originating from hematopoietic stem macrophages expressing CD11b. The dramatic increase in CD11b implied that TP/LPS co-treatment mainly recruited blood-derived monocytes to the liver and significantly increased hepatic macrophages. Additionally, macrophages can also be divided into two different and opposing functional phenotypes, traditionally named M1-type (pro-inflammatory type) and M2-type (anti-inflammatory type). The alteration in macrophage polarization determines the outcome of several liver diseases (Kazankov et al., 2019). Our result revealed that TP alone had no obvious effect on M1 macrophages (*IL-12b*, *Nos2*, and *IL1b*) as well as M2 macrophage markers (*Mrc1*, *Arginase1*, and *Retnla*). LPS treatment partly increased the M1 macrophage markers while having little effect on M2 macrophage markers, which was consistent with the published results (Wang et al., 2018). Unexpectedly, TP/LPS co-treatment boosted the expression of M1 macrophage markers while did not affect M2 macrophage markers (Figures 2E–J). Overall, our result implied that TP/LPS co-treatment increased hepatic macrophage numbers mainly from blood-derived monocytes. Moreover, macrophages might aggravate the liver injury via skewing macrophages to M1 pro-inflammatory type.

### Depletion of macrophages alleviated TP-induced indirect hepatotoxicity

**FIGURE 3:**
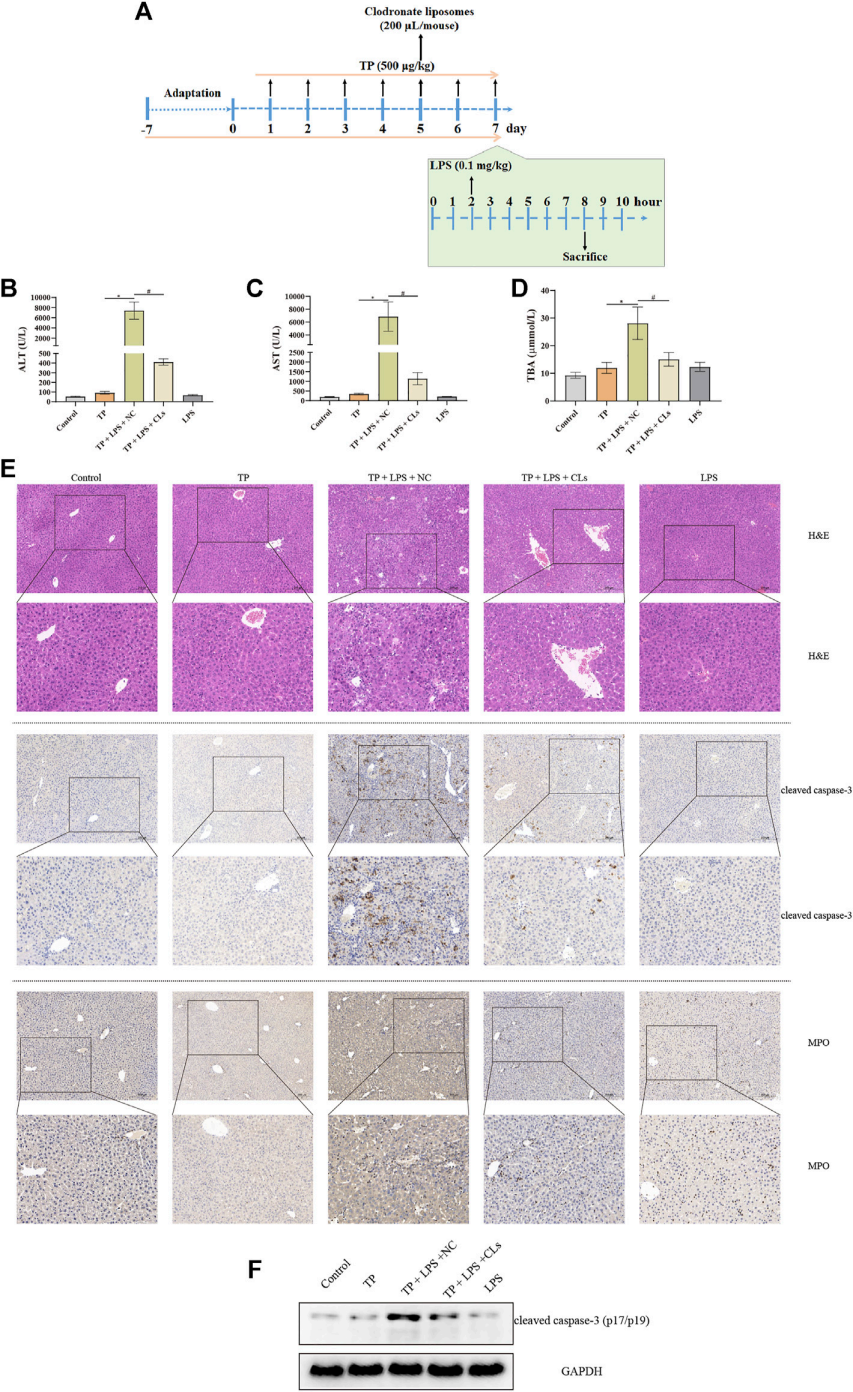
Depletion of macrophages alleviated TP-induced indirect hepatotoxicity, accompanied by relieving inflammatory reaction and hepatocyte apoptosis. **(A)** Schematic presentation of the experimental procedure to investigate the effect of depletion of macrophages in TP/LPS-induced hepatotoxicity. **(B–D)** Levels of serum ALT, AST, and TBA in mice having liver macrophages and then treatment with TP and LPS (*n* = 8). **(E)** Pictures of liver tissue sections stained by H&E (top panels), analyzed by IHC for apoptosis marker cleaved caspase-3 (middle panels) or analyzed by inflammatory marker MPO (bottom panels) (×200). **(F)** Representative protein bands of cleaved caspase-3 after the depletion of macrophages, with GAPDH as the loading control. Results were expressed as means ± SEM. Statistical analysis was performed using One-way ANOVA followed by Tukey’s Multiple Comparison Test. *p* < 0.05 was considered to be statistically different.

**FIGURE 4:**
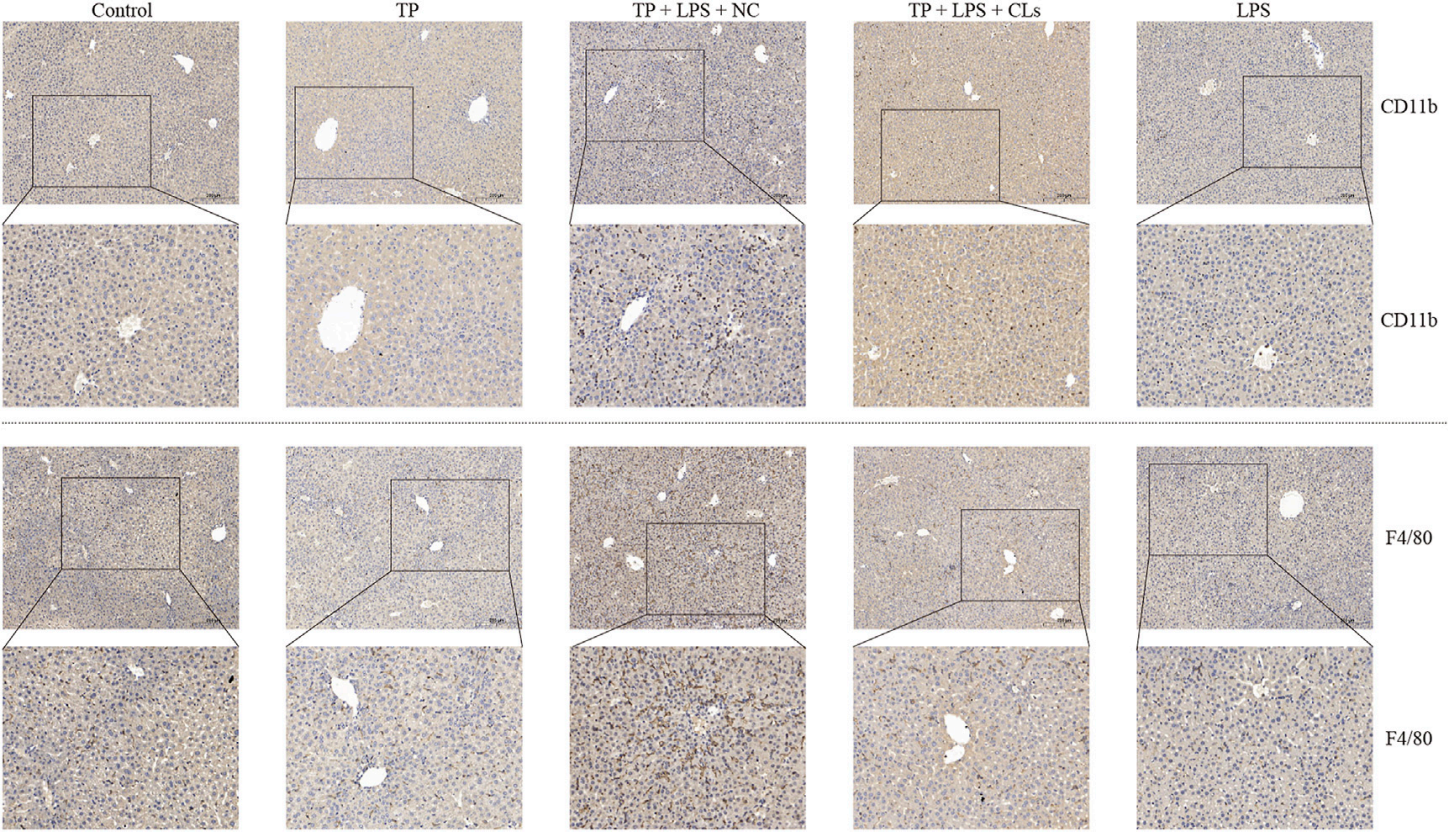
Clodronate liposomes administration constrained the recruitment of macrophages to the liver. Pictures of liver tissue sections were analyzed by IHC for macrophage markers CD11b (top panels) and F4/80 (bottom panels) (×200).

To investigate the effect of hepatic macrophages in TP-induced indirect hepatotoxicity, we depleted macrophages via clodronate liposomes, which can be phagocytized by macrophages and ultimately result in macrophage death. We injected the clodronate liposomes and its negative control (200 μl/mouse) on day 5 after TP administration, finding that depletion of macrophages apparently tempered the liver injury induced by TP/LPS co-treatment, as reflected by decreasing serum ALT, AST, and TBA levels (Figure 3B–C). Furthermore, H&E staining revealed that clodronate liposomes pretreatment alleviated the morphological changes after TP/LPS administration. Western blot and IHC staining of cleaved caspase-3 and MPO implied that clodronate liposomes weakened the apoptosis rate and the recruitment of inflammatory cells (Figures 3E,F). We additionally confirmed that clodronate liposomes pretreatment reduced the recruitment of macrophages to the liver, reflected by the decrease in CD11b and F4/80 positive cells (Figure 4). Thus, depletion of hepatic macrophages observably diminished the hepatotoxicity of TP/LPS accompanied by relieving hepatic apoptosis, necrosis, and inflammatory reactions.

### Malfunctioning of macrophages in TP-induced indirect hepatotoxicity

**FIGURE 5:**
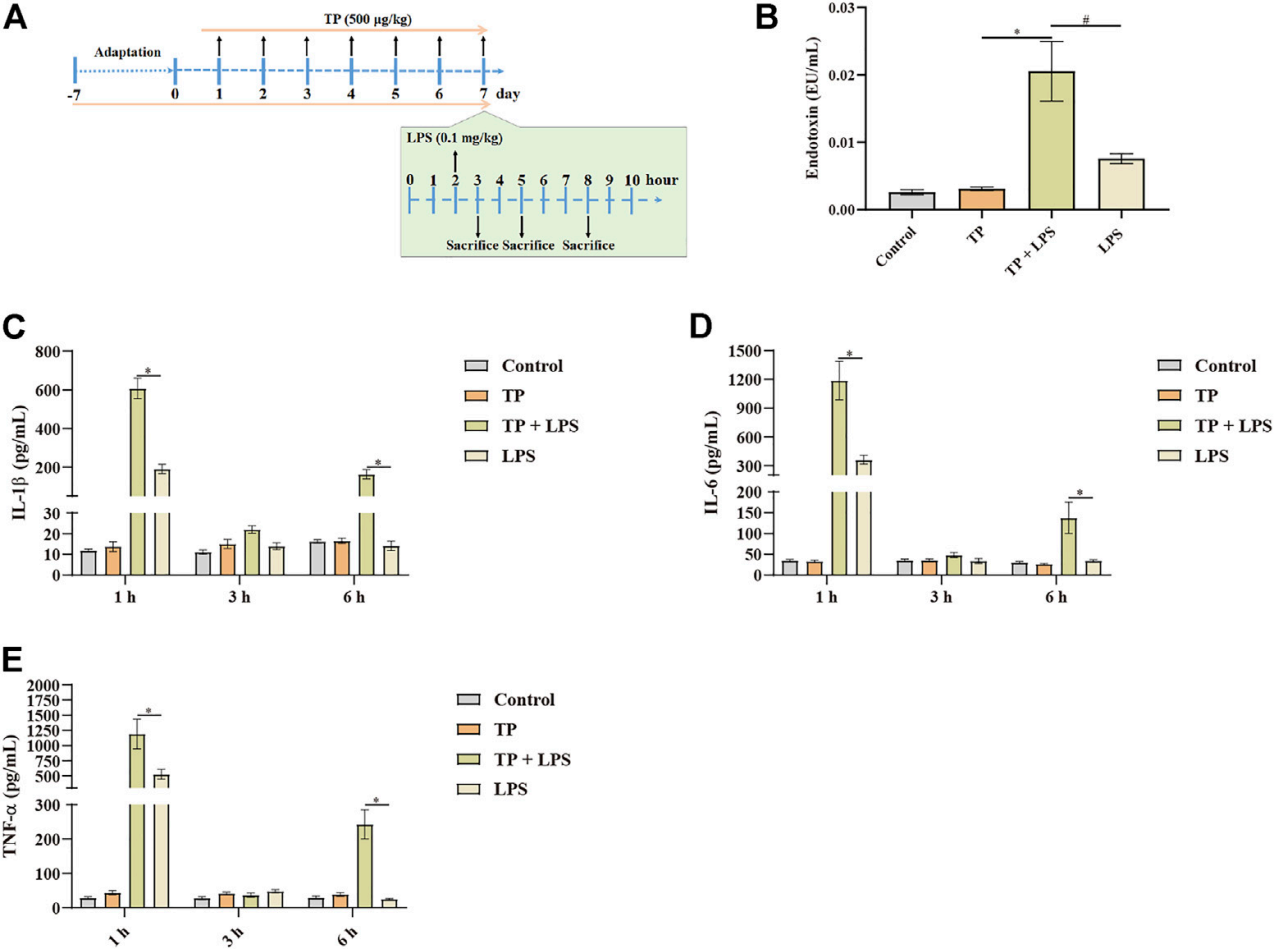
Malfunctioning of macrophages in TP-induced indirect hepatotoxicity. **(A)** Schematic presentation of the experimental procedure to investigate the time-dependent release of inflammatory factors in mice treated with TP (500 μg/kg) and LPS (0.1 mg/kg). **(B)** Serum endotoxin content in mice treated with TP and LPS (*n* = 8) 6 h after LPS injection. **(C–E)** Changes in serum TNF-α, IL-6, and IL-1β in mice after the treatment of TP and LPS (*n* = 8). Results were expressed as means ± SEM. Statistical analysis was performed using One-way ANOVA and Two-way ANOVA followed by Tukey’s Multiple Comparison Test. *p* < 0.05 was considered to be statistically different.

Although hepatic macrophages had been proved to involve in TP/LPS-induced hepatotoxicity, the way through which macrophages participated in the indirect hepatotoxicity of TP remained unclear. Hepatic macrophages functioned for phagocytosis, recognition of danger signals, the release of inflammatory factors, antigen presentation, and orchestrating immune response (Guillot and Tacke, 2019). Among them, phagocytosis and inflammatory reactions are two main functions of hepatic macrophages. When hepatocytes are undergoing necrosis, they may release death-associated molecular-pattern molecules, such as IL-1α, HMGB1, mtDNA, and HSPs, which further recruit neutrophils, dendritic cells, NK cells, and others for promoting inflammatory reactions (Gong et al., 2020). Thus, it is irrational to detect the effect of TP on the inflammatory function of macrophages 6 h after LPS administration. In our previous experiment, we found that TP/LPS co-treatment could not induce liver damage 1 h and 3 h after LPS treatment. Additionally, LPS promoted the releasing of inflammation factors in a time-dependent manner in previous reports (Shaw et al., 2007; Lu et al., 2012; Filliol et al., 2017). Thus, we detected IL-1β, IL-6, and TNF-α 1 h, 3 h, and 6 h after LPS application (Figure 5A). Our results proved that LPS increased serum IL-1β, TNF-α, and IL-6, 1 h after LPS application while these pro-inflammatory factors returned to normal subsequently. To our surprise, TP pretreatment increased the production of pro-inflammatory factors upon LPS exposure, compared with the LPS treated group (Figures 5C–E). These results proved that TP pretreatment changed the ability of macrophages to secrete inflammatory factors. Moreover, we detected the serum endotoxin levels, discovering that serum endotoxin content was higher in TP/LPS co-treatment group compared with LPS treated alone, which might also be one reason for the boost increase in pro-inflammatory factors (Figure 5B). Physiologically, endotoxin can be phagocytized in macrophages. The accumulation of endotoxin in serum in the TP/LPS co-treatment group implied TP pretreatment weakened the phagocytic capacity of hepatic macrophages. Overall, the results in this part proved that TP pretreatment affected the inflammatory secretion and phagocytic capacity of hepatic macrophages, which might be the main reason for TP/LPS-induced hepatotoxicity.

### Macrophage MerTK might severed as the new target for TP-induced indirect hepatotoxicity

**FIGURE 6:**
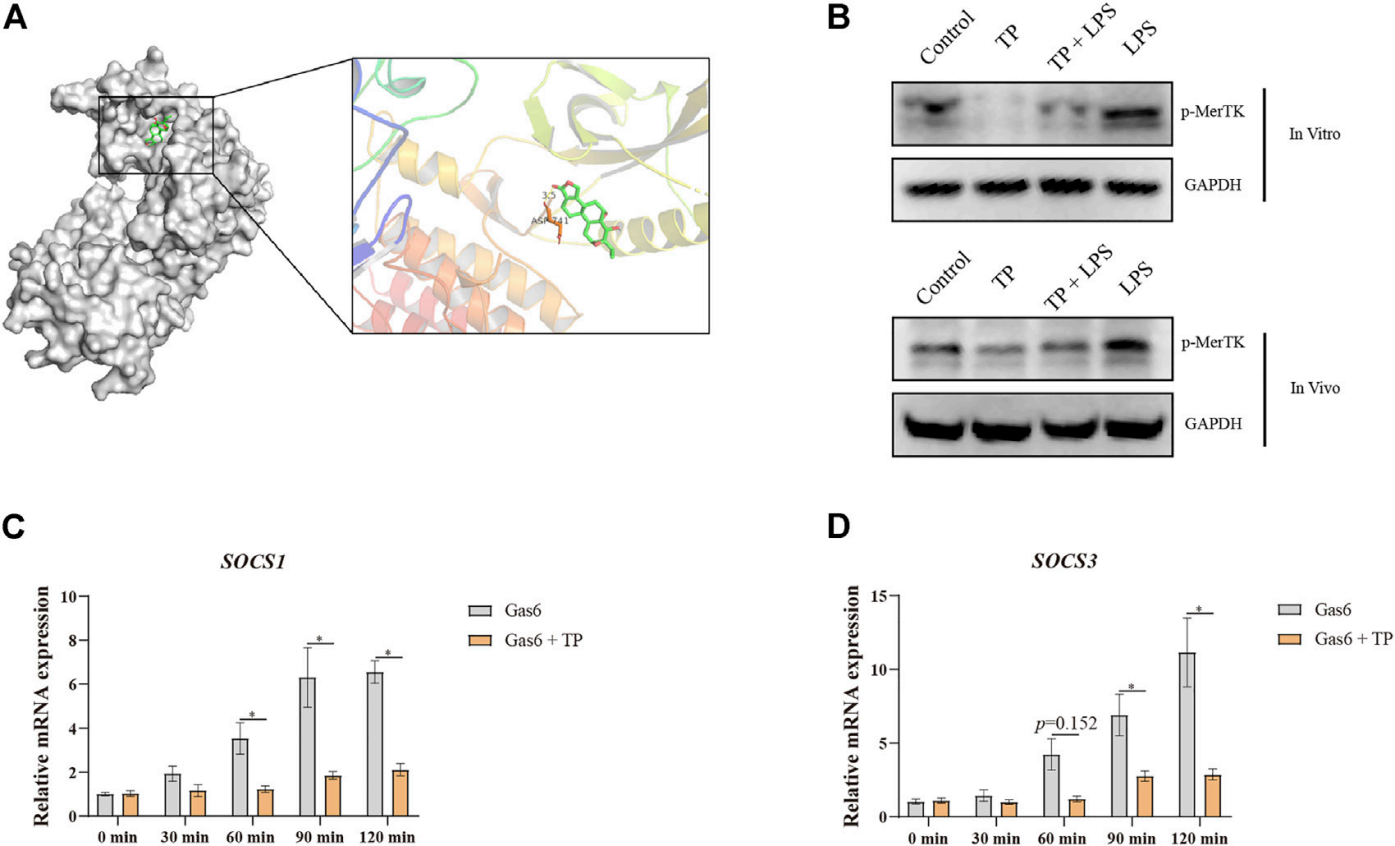
Macrophage MerTK might be the new target for TP. **(A)** Molecular docking of TP and MerTK (RCSB code: 7VAX) visualized with PyMOL. **(B)** Representative protein bands of p-MerTK in RAW264.7 cells (top panels) and mice (bottom panels) after TP and LPS treatment, with GAPDH as the loading control. The procedure of *in vivo* experiment was illustrated in Figure 1. In the *in vitro* experiment, TP (20 nmol/L) was preincubated 2 h before LPS (50 ng/ml). Twenty-4 hours after LPS, cells were collected for western blot analysis. **(C,D)** RAW264.7 cells were preincubated with TP (20 nmol/L) for 2 h and then stimulated with Gas6 (50 nmol/L). Cells were harvested at the indicated time points. The relative mRNA levels of *SOCS1* and *SOCS3* were detected by qPCR with *Gapdh* as the internal control (*n* = 4). Results were expressed as means ± SEM. Statistical analysis was performed using Two-way ANOVA followed by Tukey’s Multiple Comparison Test. *p* < 0.05 was considered to be statistically different.

To further investigate the target of TP on the macrophage, we used reversed docking technology. TP was used as the ligand to bind to the protein structure in RCSB Protein Data Bank (https://www.pdbus.org/). We selected the top 100 proteins and tried to illustrate their bioactivity with macrophages. Among them, we found MerTK, a receptor tyrosine kinase that could regulate macrophage inflammatory secretion, phagocytosis, and polarization. MerTK restricted inflammatory secretion, promoted macrophage phagocytosis, and also skewed macrophages polarization to M2 type (Cai et al., 2016; Grabiec et al., 2018; Triantafyllou et al., 2018; Zhou et al., 2020; Vago et al., 2021; Wu et al., 2021). The binding of LPS to TLR4 led to the production of inflammatory factors, which in turn up-regulated MerTK. MerTK upregulation could selectively induce the production of SOCS1 and SOCS3, ultimately blocking the inflammatory response (O'Neill, 2007; Rothlin et al., 2007). The molecule docking results implied that TP might bind to MerTK (Asp741), which is the key amino acid residue for MerTK activation (Figure 6A). *In vivo* and *in vitro* western blot analysis proved that LPS evaluated the expression of p-MerTK, while TP inhibited MerTK phosphorylation, suggesting that TP could inhibit the bioactivity of MerTK (Figure 6B). Moreover, we selected the widely accepted MerTK ligand, Growth arrest-specific gene 6 (Gas6), to investigate the effect of TP on Gas6-induced MerTK activation according to the published research. We found that Gas6 time-dependent increase in *SOCS1* and *SOCS3* transcription happened within 120 min, while TP pretreatment restrained this process (Figures 6C,D). Thus, we proved that TP inhibited the activation of MerTK upon LPS and Gas6 stimulation, the binding of TP to MerTK (Asp741) might be responsible for these processes.

## Discussion

Indirect hepatotoxicity, mainly generated from the use of drugs clinically, is a type of toxicity secondary to the pharmacological effects of drugs, providing a new direction for rational interpretation of clinically relevant drug-induced liver injury. For example, Risperidone and Haloperidol may increase body weight and the incidence of fatty liver disease (Kumra et al., 1997). The application of anticancer chemotherapeutic agents may reactivate hepatitis B (Di Bisceglie et al., 2015). Moreover, antineoplastic checkpoint inhibitors and TNF-α antagonists may induce immune-mediated liver injury due to their immune regulatory effects. Although indirect hepatotoxicity is gradually accepted by researchers, the underlying mechanisms and the animal models to investigate drug-induced indirect liver injury are still elusive. Previously, we proposed that the inhibition of c-FLIP by TP is responsible for TP-induced indirect hepatotoxicity. Additionally, the deficiency of c-FLIP increased the hepatocyte hypersensitivity to TNF-α. Thus, it is rational to explore the interaction between TNF-α secreting immune cells and hepatocytes.

We speculated that hepatic macrophages, which are the main source of TNF-α in LPS stimulation, might participate in TP-induced indirect liver injury. To prove our hypothesis, we detected the F4/80, the marker of macrophages, finding that TP/LPS co-treatment increased the number of macrophages in the liver. Additionally, CD11b, the marker of monocyte-derived macrophages also dramatically increased, revealing that TP/LPS co-treatment recruited blood-derived monocytes to the liver to participate in inflammatory responses. Macrophage polarization has been proved to hold a central position in a variety of liver diseases, including acetaminophen-induced liver injury, liver fibrosis, as well as non-alcoholic fatty liver disease (Luo et al., 2017; Zhang et al., 2019; Li et al., 2020; Shu et al., 2021). The ambivalent function of macrophages in different models of liver diseases reflects that there exist different subsets of macrophages that show opposing bio-activities. Generally, macrophages can be divided into proinflammatory M1 and immunoregulatory M2 subtypes. M1 macrophages function to release the pro-inflammatory factors, and clearance of bacteria, and viruses. In contrast, M2 macrophages could accelerate wound healing and exert anti-inflammatory activity. In this study, we found that LPS alone promoted macrophage polarization towards M1. However, TP/LPS significantly increased M1 macrophage markers, suggesting that M1 macrophages might recruit massive immune cells which were responsible for liver injury induced by TP/LPS co-treatment. To prove this, we depleted liver macrophages with clodronate liposomes. Clodronate liposomes could be phagocytized by macrophages, ultimately promoting macrophage death when injected *in vivo*. As expected, pre-treatment with clodronate liposomes almost completely alleviated the liver injury induced by TP/LPS. We supposed that cutting off the production of TNF-α was the main reason for this phenomenon (Yuan et al., 2020).

Next, we tried to investigate whether TP-treatment could disrupt the normal function of hepatic macrophages. Macrophage phagocytosis has been recognized as an important function in relieving inflammatory reactions (Underhill and Goodridge, 2012; Kourtzelis et al., 2020). Moreover, liver macrophages recognize liver injury and subsequently trigger their activation, ultimately promoting the release of inflammatory chemokines and cytokines, and also the recruitment of inflammatory cells. In this study, we detected inflammatory cytokine secretion and phagocytosis of LPS by macrophages (Figures 5B–E). To our surprise, a time-dependent *in vivo* experiment found that TP/LPS significantly promoted the release of IL-1β, IL-6, and TNF-α at the early time treatments after LPS exposure, in comparison with LPS treatment alone. Under physiological conditions, a low dose of LPS was taken by hepatic macrophages. The structure of lipid A in LPS was hydrolyzed by acyloxyacyl hydrolase in the macrophages, finally inactivating LPS (Shao et al., 2007). In our experiment, we detected plasma endotoxin concentration and found that TP pre-treatment repressed the degradation of LPS. These results firmly implied the macrophage malfunction in TP-induced indirect hepatotoxicity.

Then, we used reverse docking to find the target of TP in macrophages. The molecular docking results proved that TP might bind to MerTK, a newly found TAM receptor that is responsible for the maintenance of liver immune tolerance via inhibiting the production of inflammatory factors upon LPS stimulation and promoting macrophage efferocytic capacity. Existing studies proved that loss of MerTK amplified the inflammatory response of LPS and macrophage MerTK promoted the dissolution of inflammation in acute liver failure (O'Neill, 2007; Cai et al., 2016; Triantafyllou et al., 2018; Bernsmeier et al., 2015). Moreover, MerTK is the key receptor to maintain macrophage efferocytic capacity and its deficiency impairs the clearance of dead cells by macrophages (Caberoy et al., 2010; Grabiec et al., 2018; Gerlach et al., 2021). MerTK has also been reported to increase the M1 to M2 ratio in tumor-associated macrophages and microglial cells (Oliva et al., 2021; Wu et al., 2021). Also, MerTK deficiency in macrophages increases the sensitivity of the liver to LPS (Triantafyllou et al., 2018). These researches firmly implied that MerTK might be the true target of TP in macrophages. Our results revealed that TP inhibited the up-regulation of p-MerTK upon LPS *in vivo* and *in vitro*, which was in accordance with the molecular docking results. On binding to MerTK, Gas6 has been proved to activate the SOCS1/SOCS3 pathway. Our results also found that TP pre-treatment hindered the activation of SOCS1/SOCS3 when treated with Gas6, implying that TP inhibited the activation of MerTK. However, the binding site of TP with MerTK still needs to be proved. Also, the efferocytic capacity of MerTK relies on cGAS/STING/TBK1/IFN-β pathway, and the inflammation regulatory function of MerTK is dependent on the STAT1/SOCS1/SOCS3 pathway (O'Neill, 2007). Whether the disruption of these two pathways actually participates in TP-induced indirect hepatotoxicity needs to be further investigated. Additionally, whether the inhibition of MerTK increases the risk of indirect hepatotoxicity needs more experimental results.

## Conclusion

**FIGURE 7:**
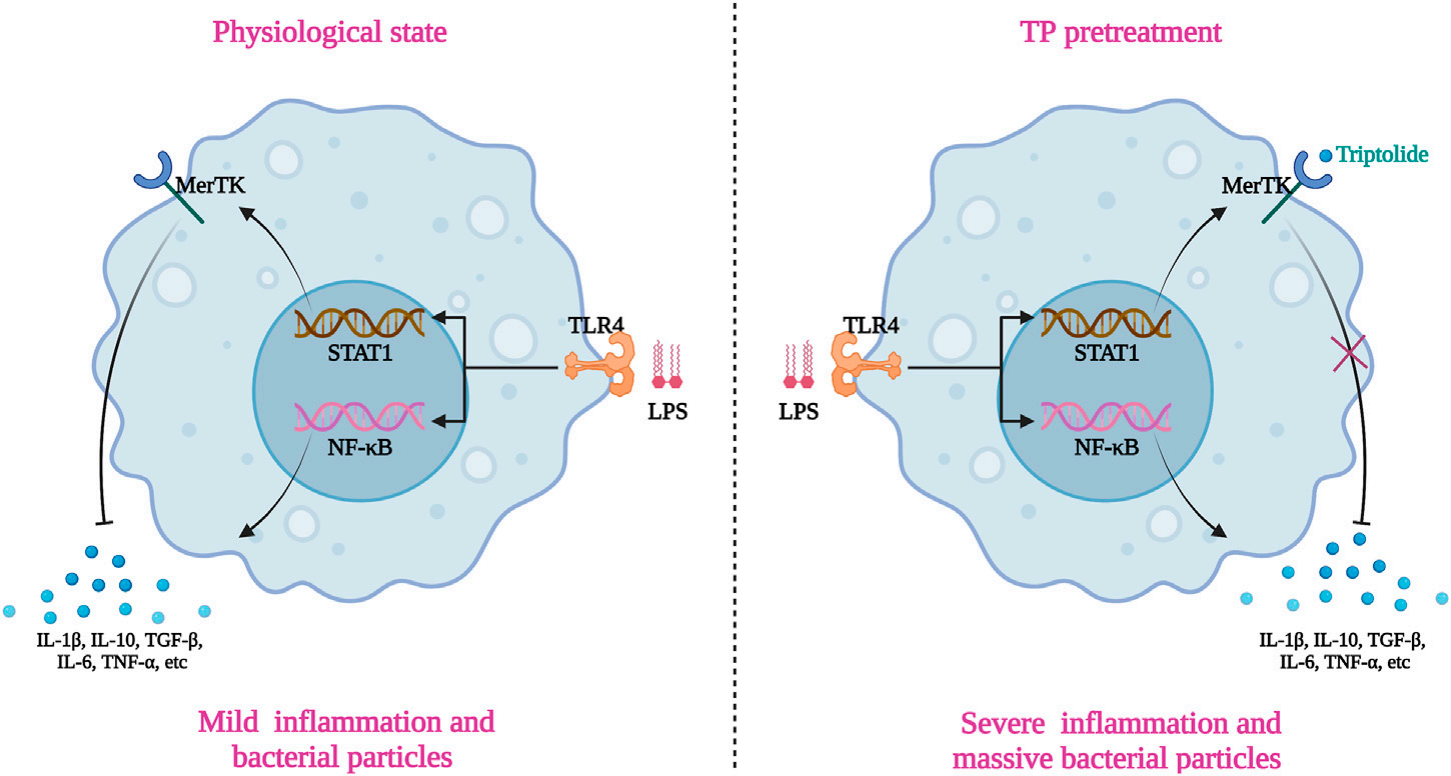
Schematic presentation indicated the suggested mechanisms by which macrophages malfunction in TP/LPS-induced indirect hepatotoxicity. The graphic was created with BioRender.com (aggrement number: XI24A5DAOR).

TP broke the immune tolerance state of hepatic macrophage via binding and inactivate MerTK, which limited the uptake of bacterial particles (including LPS) and intense the production of pro-inflammatory factors upon LPS stimulation (Figure 7). The malfunction of macrophages together with cytoprotective protein c-FLIP deficiency in hepatocyte proved in our previous research, ultimately resulting in TP-induced indirect hepatotoxicity (Yuan et al., 2020). Targeting hepatic macrophages might be the alternative option for the treatment of indirect hepatotoxicity of TP.

## Data Availability

The raw data supporting the conclusions of this article will be made available by the authors, without undue reservation.

## Supplementary material

Supplementary Figure S1 Graph depicting percentage of liver with cleaved caspase-3 (A), MPO (B), CD11b (C), and F4/80 (D) positive cells in mice treated with TP (500 μg/kg), LPS (0.1 mg/kg) or both. Results were expressed as means ± SEM. Statistical analysis was performed using One-way ANOVA followed by Tukey’s Multiple Comparison Test. p < 0.05 was considered to be statistically different.

Supplementary Figure S2 Graph depicting percentage of liver with cleaved caspase-3 (A), MPO (B), CD11b (C), and F4/80 (D) positive cells in mice treated with TP (500 μg/kg), LPS (0.1 mg/kg), Clodronate liposomes (200 μL/mouse) or its negative control. Results were expressed as means ± SEM. Statistical analysis was performed using One-way ANOVA followed by Tukey’s Multiple Comparison Test. p < 0.05 was considered to be statistically different.

## References

[B1] Bernsmeier C. Pop O. T. Singanayagam A. Triantafyllou E. Patel V. C. Weston C. J. (2015). Patients with acute-on-chronic liver failure have increased numbers of regulatory immune cells expressing the receptor tyrosine kinase MERTK. Gastroenterology 148, 603–615. 10.1053/j.gastro.2014.11.045 25479139

[B2] Caberoy N. B. Zhou Y. Li W. (2010). Tubby and tubby-like protein 1 are new MerTK ligands for phagocytosis. Embo J. 29, 3898–3910. 10.1038/emboj.2010.265 20978472 PMC3020645

[B3] Cai B. Dongiovanni P. Corey K. E. Wang X. Shmarakov I. O. Zheng Z. (2020). Macrophage MerTK promotes liver fibrosis in nonalcoholic steatohepatitis. Cell Metab. 31, 406–421. 10.1016/j.cmet.2019.11.013 31839486 PMC7004886

[B4] Cai B. Kasikara C. Doran A. C. Ramakrishnan R. Birge R. B. Tabas I. (2018). MerTK signaling in macrophages promotes the synthesis of inflammation resolution mediators by suppressing CaMKII activity. Sci. Signal. 11, eaar3721. 10.1126/scisignal.aar3721 30254055 PMC6171110

[B5] Cai B. Thorp E. B. Doran A. C. Subramanian M. Sansbury B. E. Lin C. S. (2016). MerTK cleavage limits proresolving mediator biosynthesis and exacerbates tissue inflammation. Proc. Natl. Acad. Sci. U. S. A. 113, 6526–6531. 10.1073/pnas.1524292113 27199481 PMC4988577

[B6] Challa T. D. Wueest S. Lucchini F. C. Dedual M. Modica S. Borsigova M. (2019). Liver ASK1 protects from non-alcoholic fatty liver disease and fibrosis. EMBO Mol. Med. 11, e10124. 10.15252/emmm.201810124 31595673 PMC6783644

[B7] Chen D. Ni H. M. Wang L. Ma X. Yu J. Ding W. X. (2019). p53 up-regulated modulator of apoptosis induction mediates acetaminophen-induced necrosis and liver injury in mice. Hepatology 69, 2164–2179. 10.1002/hep.30422 30552702 PMC6461480

[B8] Di Bisceglie A. M. Lok A. S. Martin P. Terrault N. Perrillo R. P. Hoofnagle J. H. (2015). Recent US food and drug administration warnings on Hepatitis B reactivation with immune-suppressing and anticancer drugs: Just the tip of the iceberg? Hepatology 61, 703–711. 10.1002/hep.27609 25412906 PMC5497492

[B9] Filliol A. Piquet-Pellorce C. Raguenes-Nicol C. Dion S. Farooq M. Lucas-Clerc C. (2017). RIPK1 protects hepatocytes from Kupffer cells-mediated TNF-induced apoptosis in mouse models of PAMP-induced hepatitis. J. Hepatol. 66, 1205–1213. 10.1016/j.jhep.2017.01.005 28088582

[B10] Gerlach B. D. Ampomah P. B. Yurdagul A. Jr. Liu C. Lauring M. C. Wang X. (2021). Efferocytosis induces macrophage proliferation to help resolve tissue injury. Cell Metab. 33, 2445–2463.e8. 10.1016/j.cmet.2021.10.015 34784501 PMC8665147

[B11] Gong T. Liu L. Jiang W. Zhou R. (2020). DAMP-sensing receptors in sterile inflammation and inflammatory diseases. Nat. Rev. Immunol. 20, 95–112. 10.1038/s41577-019-0215-7 31558839

[B12] Grabiec A. M. Goenka A. Fife M. E. Fujimori T. Hussell T. (2018). Axl and MerTK receptor tyrosine kinases maintain human macrophage efferocytic capacity in the presence of viral triggers. Eur. J. Immunol. 48, 855–860. 10.1002/eji.201747283 29400409 PMC6001567

[B13] Graham D. K. DeRyckere D. Davies K. D. Earp H. S. (2014). The TAM family: Phosphatidylserine sensing receptor tyrosine kinases gone awry in cancer. Nat. Rev. Cancer 14, 769–785. 10.1038/nrc3847 25568918

[B14] Guillot A. Tacke F. (2019). Liver macrophages: Old dogmas and new insights. Hepatol. Commun. 3, 730–743. 10.1002/hep4.1356 31168508 PMC6545867

[B15] Hasnat M. Yuan Z. Naveed M. Khan A. Raza F. Xu D. (2019). Drp1-associated mitochondrial dysfunction and mitochondrial autophagy: A novel mechanism in triptolide-induced hepatotoxicity. Cell Biol. Toxicol. 35, 267–280. 10.1007/s10565-018-9447-8 30542779

[B16] Hasnat M. Yuan Z. Ullah A. Naveed M. Raza F. Baig M. M. F. A. (2019). Mitochondria-dependent apoptosis in triptolide-induced hepatotoxicity is associated with the Drp1 activation. Toxicol. Mech. Methods 30, 124–133. 10.1080/15376516.2019.1669247 31557070

[B17] Holtzhausen A. Harris W. Ubil E. Hunter D. M. Zhao J. Zhang Y. (2019). TAM family receptor kinase inhibition reverses MDSC-mediated suppression and augments anti-PD-1 therapy in melanoma. Cancer Immunol. Res. 7, 1672–1686. 10.1158/2326-6066.CIR-19-0008 31451482 PMC6943983

[B18] Hoofnagle J. H. Bjornsson E. S. (2019). Drug-induced liver injury - types and phenotypes. N. Engl. J. Med. 381, 264–273. 10.1056/NEJMra1816149 31314970

[B19] Ju C. Tacke F. (2016). Hepatic macrophages in homeostasis and liver diseases: From pathogenesis to novel therapeutic strategies. Cell. Mol. Immunol. 13, 316–327. 10.1038/cmi.2015.104 26908374 PMC4856798

[B20] Kasikara C. Davra V. Calianese D. Geng K. Spires T. E. Quigley M. (2019). Pan-TAM tyrosine kinase inhibitor BMS-777607 enhances anti–PD-1 mAb efficacy in a murine model of triple-negative breast cancer. Cancer Res. 79, 2669–2683. 10.1158/0008-5472.CAN-18-2614 30877108

[B21] Kazankov K. Jørgensen S. M. D. Thomsen K. L. Møller H. J. Vilstrup H. George J. (2019). The role of macrophages in nonalcoholic fatty liver disease and nonalcoholic steatohepatitis. Nat. Rev. Gastroenterol. Hepatol. 16, 145–159. 10.1038/s41575-018-0082-x 30482910

[B22] Kourtzelis I. Hajishengallis G. Chavakis T. (2020). Phagocytosis of apoptotic cells in resolution of inflammation. Front. Immunol. 11, 553. 10.3389/fimmu.2020.00553 32296442 PMC7137555

[B23] Krenkel O. Tacke F. (2017). Liver macrophages in tissue homeostasis and disease. Nat. Rev. Immunol. 17, 306–321. 10.1038/nri.2017.11 28317925

[B24] Kumra S. Herion D. Jacobsen L. K. Briguglia C. Grothe D. (1997). Case study: Risperidone-induced hepatotoxicity in pediatric patients. J. Am. Acad. Child. Adolesc. Psychiatry 36, 701–705. 10.1097/00004583-199705000-00022 9136506

[B25] Lefebvre E. Moyle G. Reshef R. Richman L. P. Thompson M. Hong F. (2016). Antifibrotic effects of the dual CCR2/CCR5 antagonist Cenicriviroc in animal models of liver and kidney fibrosis. PLoS One 11, e0158156. 10.1371/journal.pone.0158156 27347680 PMC4922569

[B26] Li M. Sun X. Zhao J. Xia L. Li J. Xu M. (2020). CCL5 deficiency promotes liver repair by improving inflammation resolution and liver regeneration through M2 macrophage polarization. Cell. Mol. Immunol. 17, 753–764. 10.1038/s41423-019-0279-0 31481754 PMC7331700

[B27] Li X. J. Jiang Z. Z. Zhang L. Y. (2014). Triptolide: Progress on research in pharmacodynamics and toxicology. J. Ethnopharmacol. 155, 67–79. 10.1016/j.jep.2014.06.006 24933225

[B28] Lu J. Jones A. D. Harkema J. R. Roth R. A. Ganey P. E. (2012). Amiodarone exposure during modest inflammation induces idiosyncrasy-like liver injury in rats: Role of tumor necrosis factor-alpha. Toxicol. Sci. 125, 126–133. 10.1093/toxsci/kfr266 21984482 PMC3243747

[B29] Luo W. Xu Q. Wang Q. Wu H. Hua J. (2017). Effect of modulation of PPAR-γ activity on Kupffer cells M1/M2 polarization in the development of non-alcoholic fatty liver disease. Sci. Rep. 7, 44612. 10.1038/srep44612 28300213 PMC5353732

[B30] Marra F. Tacke F. (2014). Roles for chemokines in liver disease. Gastroenterology 147, 577–594. 10.1053/j.gastro.2014.06.043 25066692

[B31] O'Neill L. A. (2007). TAMpering with toll-like receptor signaling. Cell 131, 1039–1041. 10.1016/j.cell.2007.11.032 18083093

[B32] Oliva M. Chepeha D. Araujo D. V. Diaz-Mejia J. J. Olson P. Prawira A. (2021). Antitumor immune effects of preoperative sitravatinib and nivolumab in oral cavity cancer: SNOW window-of-opportunity study. J. Immunother. Cancer 9, e003476. 10.1136/jitc-2021-003476 34599023 PMC8488751

[B33] Rothlin C. V. Ghosh S. Zuniga E. I. Oldstone M. B. Lemke G. (2007). TAM receptors are pleiotropic inhibitors of the innate immune response. Cell 131, 1124–1136. 10.1016/j.cell.2007.10.034 18083102

[B34] Shao B. Lu M. Katz S. C. Varley A. W. Hardwick J. Rogers T. E. (2007). A host lipase detoxifies bacterial lipopolysaccharides in the liver and spleen. J. Biol. Chem. 282, 13726–13735. 10.1074/jbc.M609462200 17322564

[B35] Shaw P. J. Hopfensperger M. J. Ganey P. E. Roth R. A. (2007). Lipopolysaccharide and trovafloxacin coexposure in mice causes idiosyncrasy-like liver injury dependent on tumor necrosis factor-alpha. Toxicol. Sci. 100, 259–266. 10.1093/toxsci/kfm218 17709330

[B36] Shu B. Zhou Y. X. Li H. Zhang R. Z. He C. Yang X. (2021). The METTL3/MALAT1/PTBP1/USP8/TAK1 axis promotes pyroptosis and M1 polarization of macrophages and contributes to liver fibrosis. Cell Death Discov. 7, 368. 10.1038/s41420-021-00756-x 34839365 PMC8627510

[B37] Skirecki T. Cavaillon J. M. (2019). Inner sensors of endotoxin - implications for sepsis research and therapy. FEMS Microbiol. Rev. 43, 239–256. 10.1093/femsre/fuz004 30844058

[B38] Stutchfield B. M. Antoine D. J. Mackinnon A. C. Gow D. J. Bain C. C. Hawley C. A. (2015). CSF1 restores innate immunity after liver injury in mice and serum levels indicate outcomes of patients with acute liver failure. Gastroenterology 149, 1896–1909. 10.1053/j.gastro.2015.08.053 26344055 PMC4672154

[B39] Tacke F. (2017). Targeting hepatic macrophages to treat liver diseases. J. Hepatol. 66, 1300–1312. 10.1016/j.jhep.2017.02.026 28267621

[B40] Triantafyllou E. Pop O. T. Possamai L. A. Wilhelm A. Liaskou E. Singanayagam A. (2018). MerTK expressing hepatic macrophages promote the resolution of inflammation in acute liver failure. Gut 67, 333–347. 10.1136/gutjnl-2016-313615 28450389 PMC5868289

[B41] Underhill D. M. Goodridge H. S. (2012). Information processing during phagocytosis. Nat. Rev. Immunol. 12, 492–502. 10.1038/nri3244 22699831 PMC5570470

[B42] Vago J. P. Amaral F. A. van de Loo F. A. J. (2021). Resolving inflammation by TAM receptor activation. Pharmacol. Ther. 227, 107893. 10.1016/j.pharmthera.2021.107893 33992683

[B43] van der Heide D. Weiskirchen R. Bansal R. (2019). Therapeutic targeting of hepatic macrophages for the treatment of liver diseases. Front. Immunol. 10, 2852. 10.3389/fimmu.2019.02852 31849997 PMC6901832

[B44] Wang L. Xu D. Li L. Xing X. Liu L. Ismail Abdelmotalab M. (2018). Possible role of hepatic macrophage recruitment and activation in triptolide-induced hepatotoxicity. Toxicol. Lett. 299, 32–39. 10.1016/j.toxlet.2018.08.017 30172865

[B45] Wang X. Jiang Z. Xing M. Fu J. Su Y. Sun L. (2014). Interleukin-17 mediates triptolide-induced liver injury in mice. Food Chem. Toxicol. 71, 33–41. 10.1016/j.fct.2014.06.004 24949944

[B46] Wen Y. Lambrecht J. Ju C. Tacke F. (2021). Hepatic macrophages in liver homeostasis and diseases-diversity, plasticity and therapeutic opportunities. Cell. Mol. Immunol. 18, 45–56. 10.1038/s41423-020-00558-8 33041338 PMC7852525

[B47] Wu H. Zheng J. Xu S. Fang Y. Wu Y. Zeng J. (2021). Mer regulates microglial/macrophage M1/M2 polarization and alleviates neuroinflammation following traumatic brain injury. J. Neuroinflammation 18, 2. 10.1186/s12974-020-02041-7 33402181 PMC7787000

[B48] Yuan Z. Hasnat M. Liang P. Yuan Z. Zhang H. Sun L. (2019). The role of inflammasome activation in Triptolide-induced acute liver toxicity. Int. Immunopharmacol. 75, 105754. 10.1016/j.intimp.2019.105754 31352325

[B49] Yuan Z. Yuan Z. Hasnat M. Zhang H. Liang P. Sun L. (2020). A new perspective of triptolide-associated hepatotoxicity: The relevance of NF- kappa B and NF- kappa B-mediated cellular FLICE-inhibitory protein. Acta Pharm. Sin. B 10, 861–877. 10.1016/j.apsb.2020.02.009 32528833 PMC7280150

[B50] Yuan Z. Zhang H. Hasnat M. Ding J. Chen X. Liang P. (2019). A new perspective of triptolide-associated hepatotoxicity: Liver hypersensitivity upon LPS stimulation. Toxicology 414, 45–56. 10.1016/j.tox.2019.01.005 30633930

[B51] Zeng X. Liu G. Peng W. He J. Cai C. Xiong W. (2020). Combined deficiency of SLAMF8 and SLAMF9 prevents endotoxin-induced liver inflammation by downregulating TLR4 expression on macrophages. Cell. Mol. Immunol. 17, 153–162. 10.1038/s41423-018-0191-z 30552382 PMC7000402

[B52] Zhang H. Yuan Z. Zhu Y. Yuan Z. Wang J. Nong C. (2022). Th17/Treg imbalance mediates hepatic intolerance to exogenous lipopolysaccharide and exacerbates liver injury in triptolide induced excessive immune response. J. Ethnopharmacol. 295, 115422. 10.1016/j.jep.2022.115422 35654348

[B53] Zhang X. Fan L. Wu J. Xu H. Leung W. Y. Fu K. (2019). Macrophage p38α promotes nutritional steatohepatitis through M1 polarization. J. Hepatol. 71, 163–174. 10.1016/j.jhep.2019.03.014 30914267

[B54] Zhao G. Elhafiz M. Jiang J. Das D. Li Z. Zhou W. (2019). Adaptive homeostasis of the vitamin D-vitamin D nuclear receptor axis in 8-methoxypsoralen-induced hepatotoxicity. Toxicol. Appl. Pharmacol. 362, 150–158. 10.1016/j.taap.2018.11.002 30419252

[B55] Zhou Y. Fei M. Zhang G. Liang W. C. Lin W. Wu Y. (2020). Blockade of the phagocytic receptor MerTK on tumor-associated macrophages enhances P2x7r-dependent STING activation by tumor-derived cGAMP. Immunity 52, 357–373. 10.1016/j.immuni.2020.01.014 32049051

