# Supplementary material for: Macrophage malfunction in Triptolide-induced indirect hepatotoxicity
Source: Front Pharmacol. 2022 Sep 26;13:981996. doi: 10.3389/fphar.2022.981996 (PMC9548637; doi:10.3389/fphar.2022.981996)
Supplement: Supplementary file 1 [file Table1.DOCX]

The original data in uploaded in jianguoyun. Please refer to the link blow:

https://www.jianguoyun.com/p/DYebB8IQuIK_Chi-sMgEIAA (访问密码：oK4ZV4)
