# Supplementary figures and images for: Macrophage malfunction in Triptolide-induced indirect hepatotoxicity
Source: Front Pharmacol. 2022 Sep 26;13:981996. doi: 10.3389/fphar.2022.981996 (PMC9548637; doi:10.3389/fphar.2022.981996)

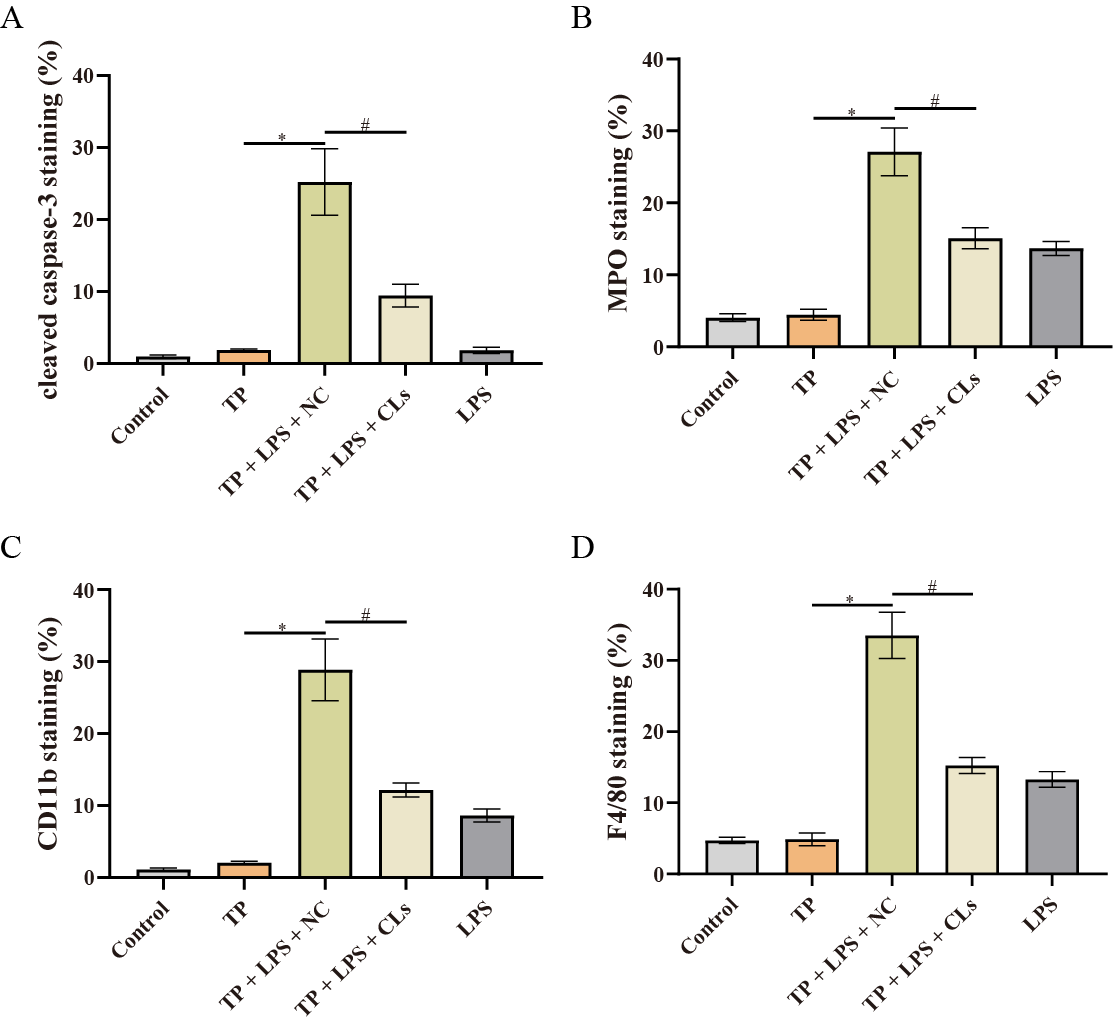

Supplement: Supplementary file 2 [file Image2.TIF]

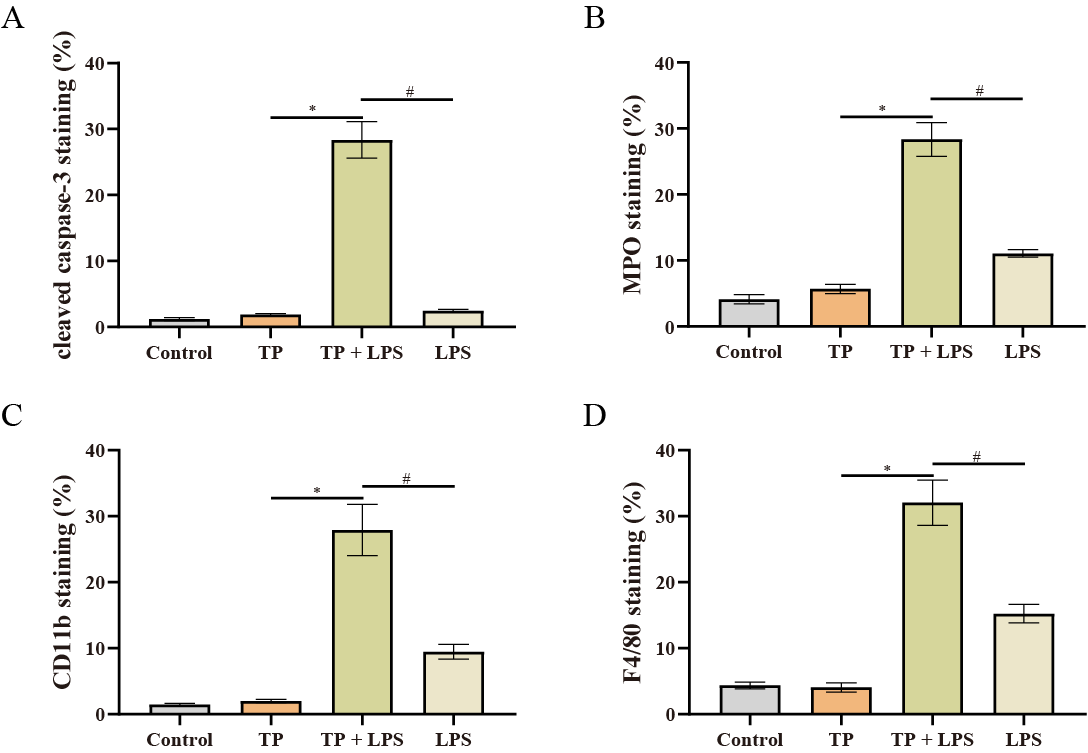

Supplement: Supplementary file 3 [file Image1.TIF]
